# Thapsigargin sensitizes human esophageal cancer to TRAIL-induced apoptosis via AMPK activation

**DOI:** 10.1038/srep35196

**Published:** 2016-10-12

**Authors:** Zhiqiang Ma, Chongxi Fan, Yang Yang, Shouyin Di, Wei Hu, Tian Li, Yifang Zhu, Jing Han, Zhenlong Xin, Guiling Wu, Jing Zhao, Xiaofei Li, Xiaolong Yan

**Affiliations:** 1 Department of Thoracic Surgery, Tangdu Hospital, The Fourth Military Medical University, 1 Xinsi Road, Xi’an 710038, China; 2 Department of Thoracic and Cardiovascular Surgery, Affiliated Drum Tower Hospital of Nanjing University Medical School, 321 Zhongshan Road, Nanjing 210008, Jiangsu, China; 3 Department of Biomedical Engineering, The Fourth Military Medical University, 169 Changle West Road, Xi’an 710032, China; 4 Department of Ophthalmology, Tangdu Hospital, The Fourth Military Medical University, 1 Xinsi Road, Xi’an 710038, China; 5 Department of Thoracic Surgery, Beijing Military General Hospital, 5 DongSi ShiTiao Road 100070, Beijing 100700, China

## Abstract

Tumor necrosis factor-related apoptosis-inducing ligand (TRAIL) is a promising anticancer agent for esophageal squamous cell carcinoma (ESCC). Forced expression of CHOP, one of the key downstream transcription factors during endoplasmic reticulum (ER) stress, upregulates the death receptor 5 (DR5) levels and promotes oxidative stress and cell death. In this study, we show that ER stress mediated by thapsigargin promoted CHOP and DR5 synthesis thus sensitizing TRAIL treatment, which induced ESCC cells apoptosis. These effects were reversed by DR5 siRNA *in vitro* and CHOP siRNA both *in vitro* and *in vivo*. Besides, chemically inhibition of AMPK by Compound C and AMPK siRNA weakened the anti-cancer effect of thapsigargin and TRAIL co-treatment. Therefore, our findings suggest ER stress effectively sensitizes human ESCC to TRAIL-mediated apoptosis via the TRAIL-DR5-AMPK signaling pathway, and that activation of ER stress may be beneficial for improving the efficacy of TRAIL-based anti-cancer therapy.

Esophageal cancer is an aggressive human malignancy and one of leading causes of death from cancer worldwide among men and women^1^. Esophageal squamous cell carcinoma (ESCC) is the predominant histological type of esophageal cancer that occurs in most Asian countries^2^. Despite recent advances, the improvements of diagnosis, treatment, and perioperative management are marginally effective. The prognosis of patients with ESCC is still poor, of which the 5-year overall survival rate ranges from 20% to 30%^3^. Therefore, it is very important to identify biological markers and novel therapeutic strategies associated with the early progression of ESCC.

The endoplasmic reticulum (ER) is the primary site for protein synthesis, folding, and trafficking^4^. In response to numerous different environmental changes, protein sensors located in the luminal face of the endoplasmic reticulum membrane activate the unfolded protein response (UPR), also referred to as ER stress^5^. Double-stranded RNA-activated protein kinase–like ER kinase (PERK), inositol requiring kinase 1 (IRE1), and activating transcription factor (ATF)-6 are three critical ER membrane-associated ER stress sensors that regulate the unfolded protein response^6^. When ER stress is persistent and unresolved, UPR will turn on specific apoptotic pathways to eliminate severely damaged cells^7^. Our previous study showed that phosphorylation of PERK and activation of CHOP attenuated protein synthesis and mediated ESCC apoptosis *in vitro* and *in vivo*^8^.

The tumor necrosis factor (TNF)-related apoptosis-inducing ligand (TRAIL), a member of the TNF family, is a potent anticancer agent due to its induction of apoptosis in a variety of tumor cell types while having minimal off target effects on normal cells^9^. Death receptor 5 (DR5, also referred to as Apo2) is one of the TRAIL receptors (TRAILRs) located on the cell surface^10^. After integrating with its ligand TRAIL and recruiting adapter proteins, DR5 forms the death-inducing signaling complex and eventually activates caspase cascades^11^. Activated caspases and proapoptotic Bcl-2 family members, such as Bax and Bad, accelerate intrinsic apoptosis^12^. Previous studies have indicated that forced expression of CHOP, caused by several agents (e.g., monensin, amiodarone, and 20(S)-ginsenoside Rg3), promotes apoptosis via the upregulation of DR5 in a variety of cancer cells^13^,^14^,^15^.

Adenosine monophosphate (AMP)-activated protein kinase (AMPK) belongs to a family of serine/threonine protein kinases that act as metabolic sensors of cellular energy change in mammalian cells^16^. Epidemiological studies found that decreased AMPK activity is accompanied by increased cancer mortality^17^. However, studies also showed that activation of AMPK signaling might facilitate growth inhibition and cell killing, serving as a new therapeutic target in cancer diseases^18^. Although the function of AMPK in metabolism is known, its roles in cell proliferation and survival remain poorly documented and somewhat controversial. It is believed that the conflicting effects depend on the cell type used, the cellular events following external stimuli, the duration of AMPK activation, and/or the pathways regulated downstream of AMPK. A recent study showed that increased cellular Ca^2+^ can phosphorylate and activate AMPK via activating Ca^2+^/calmodulin-dependent protein kinase kinase β, suggesting the association between AMPK activation and ER stress^19^. Furthermore, several studies found that AMPK activation can sensitize cancer cells to TRAIL-induced apoptosis^12^,^20^. These interesting observations, despite indicating that the regulation of the interaction between TRAIL sensitization and ER stress or AMPK activation represent potential anticancer mechanisms, do not provide the detailed mechanism involved in the relationship of these three factors in ESCC, which needs to be critically elucidated. In this study, we concomitantly used thapsigargin and the TRAIL as cytotoxic inducers to elucidate their anticancer effects on ESCC cellular activity. Meanwhile, we determined whether ER stress has potential application in TRAIL-mediated cancer therapy via AMPK activation.

## Results

### Inhibition of the cell viability of various human ESCCs, but not normal human esophageal epithelial cells, by thapsigargin and the TRAIL

**Figure 1:**
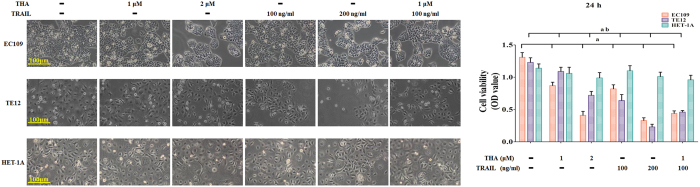
Thapsigargin and TRAIL co-treatment inhibit the viability in human ESCC cells, but not in human normal esophageal epithelial cells. Human ESCC cells (EC109 and TE12) were treated with increasing concentrations of thapsigargin (1 and 2 μM) and TRAIL (100 and 200 ng/ml) for 24 h. The cell viability was analyzed by MTT. Meanwhile, human normal esophageal epithelial cells (HET-1A) were given the same treatment as above. The cell viability is expressed as OD values. The morphology of both of the ESCC cells and normal esophageal epithelial cells were observed under an inverted phase-contrast microscope after the cells were treated for 24 h, and images were obtained. Significant cell shrinkage and a decreased cellular attachment rate were observed in the thapsigargin or TRAIL treatment groups, especially in drug combination. All of the results are expressed as the mean ± SD; n = 6. ^a^P < 0.05 *vs.* the control group in EC109 cells, ^ab^P < 0.05 *vs.* the control group in TE12 cells.

Our earlier studies have suggested that thapsigargin might synergistically enhance the anticancer activity of drugs against human ESCCs, including inhibition of tumor cell proliferation, invasion, and metastasis, as well as induction of apoptosis^8^. Moreover, TRAIL is identified as a potent tumoricidal agent with less toxicity in normal cells. However, the combinatorial effects of these two factors on ESCCs have not been explored. Thus, we first examined the sensitivity of various esophageal squamous carcinoma cell lines to TRAIL. EC109 and TE12 cells were treated with TRAIL for 24 h and then assessed for cell viability using an MTT assay. These two ESCC cell lines were found to be moderately sensitive to the TRAIL (Fig. 1). Furthermore, the viability of these ESCC cell lines was inhibited by thapsigargin in a dose-dependent manner (Fig. 1). Meanwhile, co-treatment with thapsigargin enhanced TRAIL-induced cell death dose-dependently. Unlike these ESCCs, the viability of normal human esophageal epithelial cells was not suppressed by thapsigargin and/or the TRAIL (Fig. 1), suggesting that combined treatment with thapsigargin and the TRAIL may have safe and effective cytotoxic activity against ESCCs. Additionally, the combination index (CI) was calculated according to a previous study: CI < 1 indicates synergistic activity, CI = 1 indicates additive activity, and CI > 1 indicates antagonistic activity^21^. The CIs in the two ESCC cell lines used in this study were approximately less than 1 (0.84 in EC109 and 0.80 in TE12), indicating that the combination of thapsigargin and the TRAIL exerted a synergistic effect.

### Induction of apoptosis by thapsigargin and the TRAIL in various ESCC cell lines

**Figure 2:**
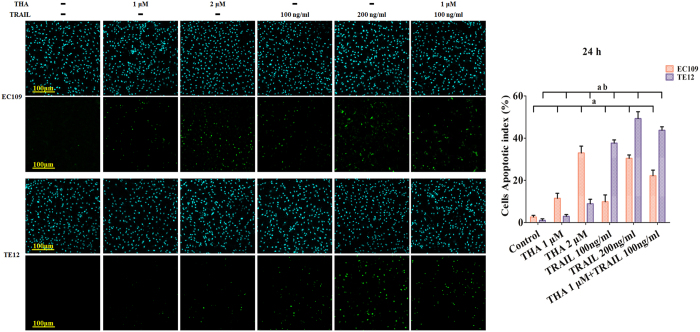
Thapsigargin and TRAIL co-treatment promote the apoptosis in human ESCC cells (24 h). After treatment, a dose-dependent increase was observed in apoptosis, particularly in combined treatment group. The upper panel showed the cell nucleus (blue) and the lower panel showed the apoptotic cells (green), respectively. All of the results are expressed as the mean ± SD; n = 6. ^**a**^P < 0.05 *vs.* the control group in EC109 cells, ^**ab**^P < 0.05 *vs.* the control group in TE12 cells.

To assess the induction of apoptosis by thapsigargin and the TRAIL, an *In Situ* Cell Death Detection Kit was used according to the manufacturer’s instructions. Thapsigargin alone induced a notable increase in apoptosis in both ESCCs, and TRAIL alone resulted in a similar increase in apoptosis in both ESCCs (Fig. 2 right). Furthermore, the combination treatment resulted in synergistic cytotoxic effects. The majority of the apoptotic cells in these two ESCCs were comparable with those in the MTT assays. Meanwhile, apoptosis induced by the combination treatment in both ESCCs was further identified by cell morphology under a BX51 fluorescence microscope (Olympus, Tokyo, Japan) (Fig. 2 left).

### Inhibition of cell migration, adhesion, and invasion induced by thapsigargin and the TRAIL in various ESCC cell lines

**Figure 3:**
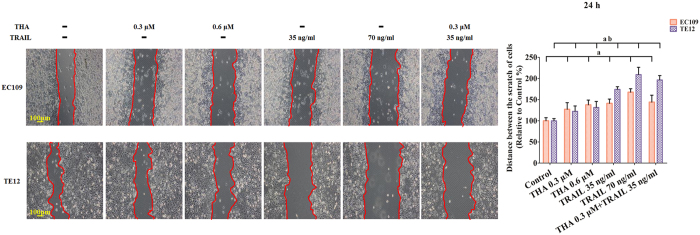
Thapsigargin and TRAIL co-treatment restrain the migration in human ESCC cells (24 h). The migratory ability of ESCC cells is expressed as the mean distance between the two sides of the scratch. The mean distance in the control group was set as 100%. The results are expressed as the mean ± SD; n = 6. ^**a**^P < 0.05 *vs.* the control group in EC109 cells, ^**ab**^P < 0.05 *vs.* the control group in TE12 cells.

**Figure 4:**
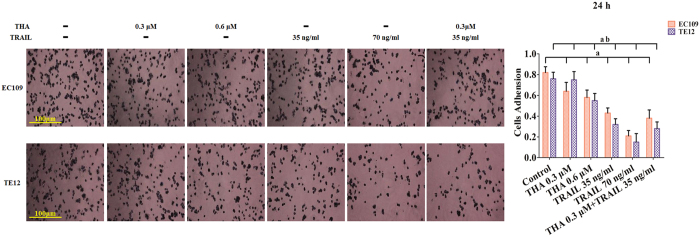
Thapsigargin and TRAIL co-treatment suppress the adhesion in human ESCC cells (24 h). The adhesion ability of ESCC cells is expressed as an adhesion ratio. The number of adherent cells in the control group was set as 100%. The results are expressed as the mean ± SD; n = 6. ^**a**^P < 0.05 *vs.* the control group in EC109 cells, ^**ab**^P < 0.05 *vs.* the control group in TE12 cells.

**Figure 5:**
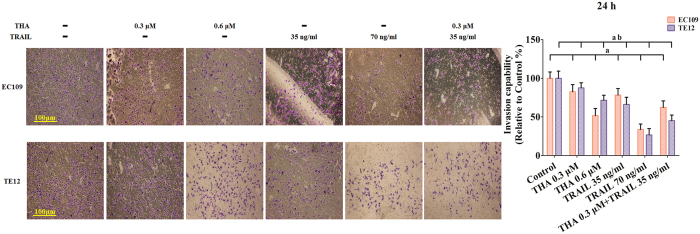
Thapsigargin and TRAIL co-treatment repress the invasion in human ESCC cells (24 h). Representative invasive capability images are shown. The invasive capability is expressed as an invasion rates. The number of invasive cells in the control group was set as 100%. The results are expressed as the mean ± SD; n = 6. ^**a**^P < 0.05 *vs.* the control group in EC109 cells,^**ab**^P < 0.05 *vs.* the control group in TE12 cells.

Considering the above results, we suspected that thapsigargin and the TRAIL might hinder cancer progression in ESCCs. To address this question, we compared the migratory and invasive ability of two ESCC cell lines using a wound-healing assay, an adhesion assay, and a transwell invasion assay. Based on our pre-experimental, the relatively low concentrations of thapsigargin (0.6 and 0.3 μM) and TRAIL (70 and 35 ng/ml) did not affect the cell viability and phosphorylation of AMPK in human ESCC cells (Supplementary Figure 1A,B). So, after incubation with thapsigargin (0.3 and 0.6 μM) for 24 h, the distance between scratches in the EC109 and TE12 cells did not reduced observably (Fig. 3), while the adhesion ratio decreased significantly in these two ESCCs (Fig. 4). Additionally, the invasion capability reflected by the transwell invasion assay was markedly suppressed (Fig. 5). Similarly, TRAIL treatment (70 and 35 ng/ml) had an anticancer effect in these two ESCC cell lines. Furthermore, co-treatment with thapsigargin and the TRAIL mediated more obviously inhibitory effects on the migratory and invasive abilities of these two ESCC cell lines (Figs 3, 4, 5). These results partly indicated that thapsigargin enhanced the TRAIL-induced reduction in metastasis abilities in ESCCs.

### Regulation of ROS generation, NADPH oxidase activity, Caspase 3 activity, Caspase 9 activity, and GSH levels in human ESCC cell lines treated with thapsigargin and the TRAIL

**Figure 6:**
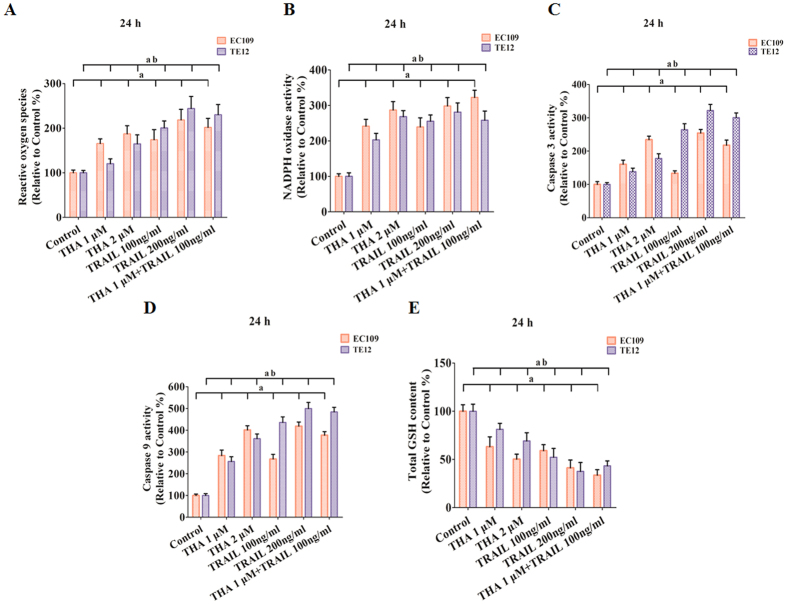
Thapsigargin and TRAIL co-treatment regulate the ROS generation, NADPH oxidase activity, Caspase 3 activity, Caspase 9 activity, and GSH levels in human ESCC cells (24 h). (**A**) ROS concentrations are shown. (**B**) NADPH oxidase activity is shown. (**C**) The intracellular Caspase 3 activity levels are shown. (**D**) The intracellular Caspase 9 activity levels are shown. (**E**) Intracellular GSH levels are shown. The three indexes in the control group were defined as 100%. The results are expressed as the mean ± SD; n = 6. ^a^P < 0.05 *vs.* the control group in EC109 cells, ^ab^P < 0.05 *vs.* the control group in TE12 cells.

To determine whether the combination of thapsigargin and the TRAIL causes intracellular oxidation, we used the specific oxidation-sensitive fluorescent dye DCFH-DA, which exhibits enhanced fluorescence intensity following the generation of reactive metabolites. Treatment with thapsigargin or the TRAIL alone for 24 h resulted in a dose-dependent increase in ROS generation in EC109 and TE12 cells (Fig. 6A). The NADPH oxidase system is now widely recognized as a key player in intracellular ROS homeostasis and as one of the major producers of ROS within the cell^22^. After administration of thapsigargin and the TRAIL, respectively, NADPH oxidase activity was increased in a dose-dependent manner (Fig. 6B). Caspase 3 activity (Fig. 6C) and Caspase 9 activity (Fig. 6D) were also significantly increased after treatment with thapsigargin or the TRAIL. GSH is the major non-protein thiol in cells and is essential for maintaining the cellular redox status. We observed a dose-dependent decrease in intracellular GSH levels after treatment (Fig. 6E). Moreover, thapsigargin combined with the TRAIL induced more distinct changes in ROS generation, NADPH oxidase activity, Caspase 3 activity, Caspase 9 activity, and GSH levels in the two ESCC cell lines. These results support the idea that thapsigargin treatment sensitized the TRAIL-induced variation of cellular redox status.

### Activation of ERS signaling is induced by thapsigargin, but not by the TRAIL, in human ESCC cell lines

**Figure 7:**
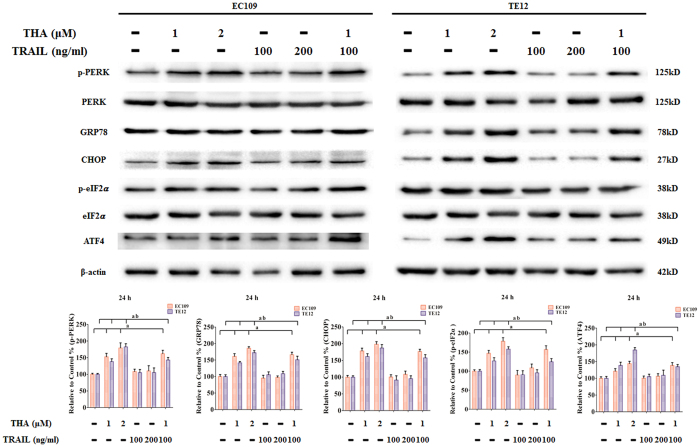
Effect of Thapsigargin and TRAIL co-treatment on ERS signaling in human ESCC cells (24 h). Representative Western blot results of p-PERK, GRP78, CHOP, p-eIF2α, and ATF4 are shown. Membranes were re-probed for β-actin expression to show that similar amounts of protein were loaded in each lane. The results are expressed as the mean ± SD; n = 6.^**a**^P < 0.05 *vs.* the control group in EC109 cells, ^**ab**^P < 0.05 *vs.* the control group in TE12 cells.

To investigate the anticancer activity of combined treatment with thapsigargin and the TRAIL on ERS signaling, ERS-related molecules were detected via western blot in the two ESCC cell lines. Western blots revealed that thapsigargin treatment improved the expression of GRP78, CHOP, and ATF4. Meanwhile, phosphorylation of PERK and eIF2α were also increased in EC109 and TE12 cells in a dose-dependent manner (Fig. 7). However, the TRAIL slightly affected the ERS signaling in ESCC cell lines, but this was not a synergistic effect.

### Induction of apoptosis with AMPK phosphorylation and DR5 upregulation induced by thapsigargin and the TRAIL in human ESCC cell lines

**Figure 8:**
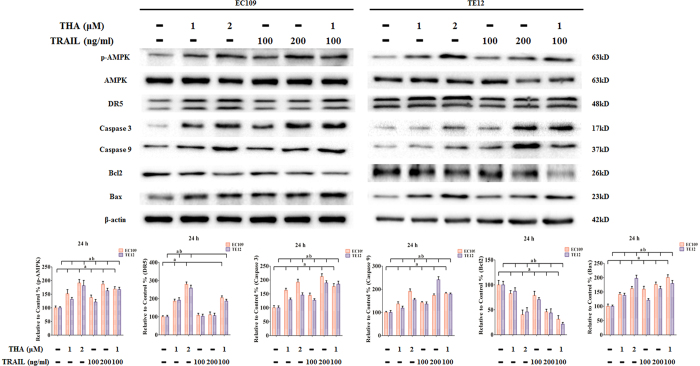
Effect of Thapsigargin and TRAIL co-treatment on AMPK phosphorylation, DR5 upregulation, and apoptosis-associated proteins in human ESCC cells (24 h). Representative Western blot results of p-AMPK, DR5, Caspase 3, Caspase 9, Bcl2 and Bax are shown. Membranes were re-probed for β-actin expression to show that similar amounts of protein were loaded in each lane. The results are expressed as the mean ± SD; n = 6. ^a^P < 0.05 *vs.* the control group in EC109 cells, ^ab^P < 0.05 *vs.* the control group in TE12 cells.

As shown in Fig. 8, we found that treatment with both thapsigargin and the TRAIL increased the phosphorylation level of AMPK, the expression of Bax, and the activation of Caspase 3 and Caspase 9 in ESCC cell lines. In contrast, Bcl2 was decreased following the same treatment. We next detected DR5, one of the TRAIL receptors. After treatment with different concentrations of thapsigargin for 24 h, DR5 protein expression was significantly upregulated. Moreover, co-treatment with thapsigargin and the TRAIL further intensified the changes of the above-mentioned proteins.

### Thapsigargin-induced cell inhibition and DR5 upregulation were mediated through induction of CHOP in human ESCC cell lines

**Figure 9:**
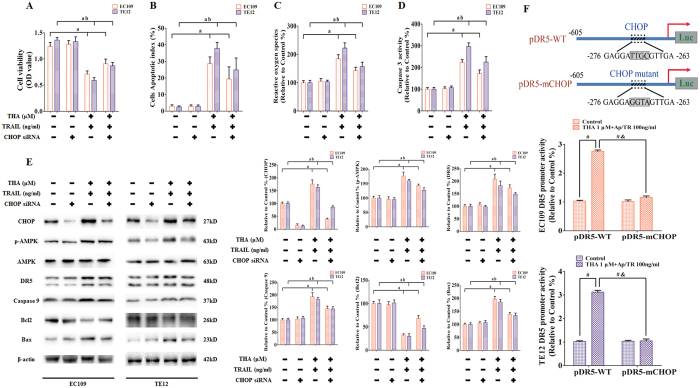
CHOP-mediated DR5 upregulation is critical for thapsigargin-stimulated TRAIL-induced apoptosis in ESCC cells. **(A)** Viability is shown as OD values. **(B)** Apoptosis is shown as apoptotic index. **(C)** ROS concentrations are shown. **(D)** The intracellular Caspase 9 activity levels are shown. The two indexes in the control group were defined as 100%. **(E)** Representative Western blot results of the key proteins, including CHOP, p-AMPK, DR5, Caspase 9, Bcl2, and Bax are shown. Membranes were re-probed for β-actin expression to show that similar amounts of protein were loaded in each lane. **(F)** Schematic diagram of the DR5 promoter constructs used for the luciferase activity assay (top). ESCC cells were transfected with pDR5-605-WT or pDR5-605-mCHOP promoter constructs and β-gal plasmid and then treated with thapsigargin (1 μM) plus TRAIL (100 ng/ml) for 24 hours and cells were lysed for luciferase assay (EC109 as middle, TE12 as bottom). The results are expressed as the mean ± SD; n = 6. ^**a**^P < 0.05 *vs.* the control siRNA group in EC109 cells, ^**ab**^P < 0.05 *vs.* the control siRNA group in TE12 cells.

Studies have shown that CHOP/GADD153 contributes to anticarcinogen-mediated upregulation of DR5, leading to sensitization of TRAIL-mediated cell death^23^,^24^. A specific siRNA was used to explore the effect of CHOP downregulation on the antitumor activity of thapsigargin with the TRAIL *in vitro*. EC109 and TE12 cells were first transfected with CHOP siRNA and then treated with thapsigargin (1 μM) and the TRAIL (100 ng/ml) for an additional 24 h. Transfection with CHOP siRNA significantly decreased the expression of the target protein in EC109 and TE12 cells (Fig. 9E). The combination of CHOP siRNA, thapsigargin, and the TRAIL significantly increased cell viability (Fig. 9A), inhibited apoptosis index (Fig. 9B), reduced the generation of ROS (Fig. 9C), and decreased Caspase 3 activity (Fig. 9D). However, CHOP siRNA alone did not affect cell viability, apoptosis index, ROS generation, or Caspase 3 activity. Instead, the phosphorylation level of AMPK, the expression levels of DR5 and Bax, and the activation of Caspase 3 were downregulated by co-treatment of the three agents, while Bcl2 was upregulated by the same treatment (Fig. 9E).

To confirm the direct involvement of CHOP in the thapsigargin-mediated transcriptional activation of DR5, we used two luciferase reporter plasmids: pDR5-WT, which contained the DR5 promoter sequence −605/+3, and pDR5-mCHOP, which contained the same promoter sequence with mutation of the potential CHOP-binding site (−281 to −261)^13^. EC109 and TE12 cells were separately transfected with these plasmids and subjected to thapsigargin treatment, and then a luciferase assay was performed. Our results demonstrated that the transcriptional activities of pDR5-WT in EC109 and TE12 cells were significantly increased by thapsigargin treatment, but the promoter activity of pDR5-mCHOP was not enhanced by the same treatment, suggesting that CHOP directly mediates the thapsigargin-induced upregulation of DR5 (Fig. 9F).

### Sensitization by thapsigargin to TRAIL-induced apoptosis is mediated by upregulation of DR5 in human ESCC cell lines

**Figure 10:**
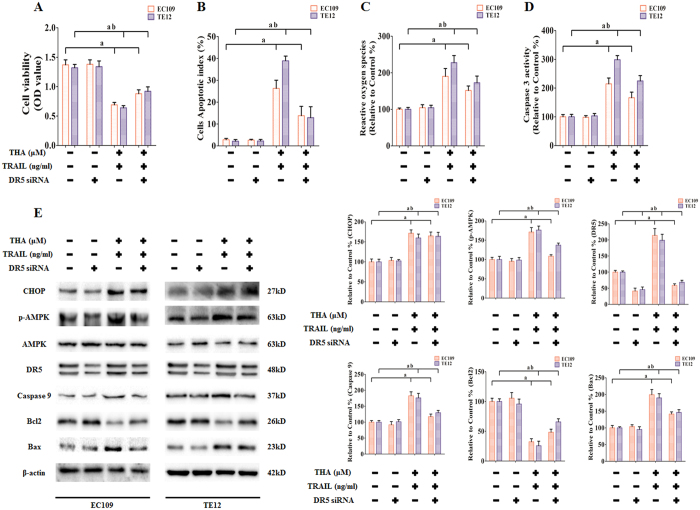
Effect of Thapsigargin and TRAIL co-treatment combined with DR5 siRNA on cell viability, apoptosis, ROS induction, Caspase 3 activity, and key proteins in human ESCC cells. (**A**) Viability is shown as OD values. (**B**) Apoptosis is shown as apoptotic index. (**C**) ROS concentrations are shown. (**D**) The intracellular Caspase 9 activity levels are shown. The two indexes in the control group were defined as 100%. (**E**) Representative Western blot results of the key proteins, including CHOP, p-AMPK, DR5, Caspase 9, Bcl2, and Bax are shown. Membranes were re-probed for β-actin expression to show that similar amounts of protein were loaded in each lane. The results are expressed as the mean ± SD; n = 6. ^a^P < 0.05 *vs.* the control siRNA group in EC109 cells, ^ab^P < 0.05 *vs.* the control siRNA group in TE12 cells.

To determine the direct roles of DR5 in thapsigargin-induced TRAIL sensitization, EC109 and TE12 cells were treated with DR5 siRNA, followed by co-treatment with thapsigargin (1 μM) and the TRAIL (100 ng/ml) for an additional 24 h. As shown in Fig. 10E, treatment with DR5 siRNA resulted in the specific inhibition of the corresponding proteins as expected. DR5 silencing antagonized thapsigargin-dependent sensitization to TRAIL in EC109 and TE12 cells, as evidenced by significantly increased cell viability (Fig. 10A), inhibited apoptosis index (Fig. 10B), reduced generation of ROS (Fig. 10C), and decreased Caspase 3 activity (Fig. 10D). However, DR5 gene silencing did not affect the expression of CHOP, but did affect AMPK phosphorylation and Bcl2 levels, and reduce Caspase 9 and Bax expression, which indicated the relationship between these proteins. These findings further suggested that thapsigargin mediated sensitization to TRAIL in ESCC cell lines through the activation of the DR5 signaling pathway.

### Role of AMPK in sensitization by thapsigargin to TRAIL-induced apoptosis in human ESCC cell lines

**Figure 11:**
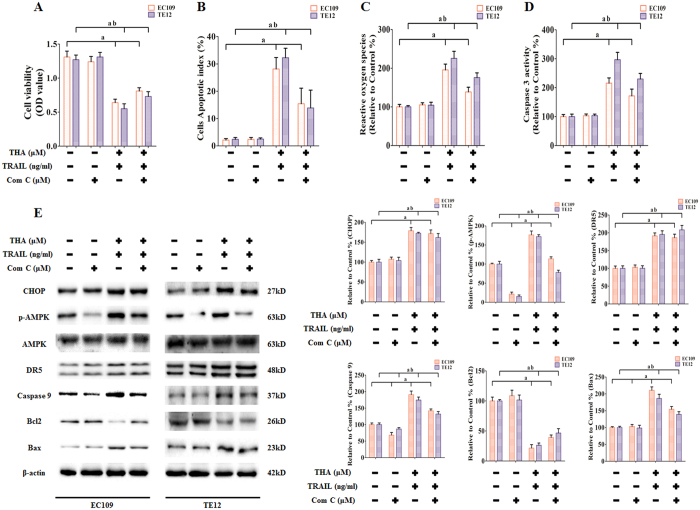
The role of AMPK activation in thapsigargin-stimulated TRAIL-induced apoptosis in ESCC cells. The ESCC cells were co-treated with Compound C (1 μM), thapsigargin (1 μM), and TRAIL (100 ng/ml) for 24 h. **(A)** Viability is shown as OD values. **(B)** Apoptosis is shown as apoptotic index. **(C)** ROS concentrations are shown. **(D)** The intracellular Caspase 9 activity levels are shown. The two indexes in the control group were defined as 100%. **(E)** Representative Western blot results of the key proteins, including CHOP, p-AMPK, DR5, Caspase 9, Bcl2, and Bax are shown. Membranes were re-probed for β-actin expression to show that similar amounts of protein were loaded in each lane. The results are expressed as the mean ± SD; n = 6. ^**a**^P < 0.05 *vs.* the control siRNA group in EC109 cells, ^**ab**^P < 0.05 *vs.* the control siRNA group in TE12 cells.

**Figure 12:**
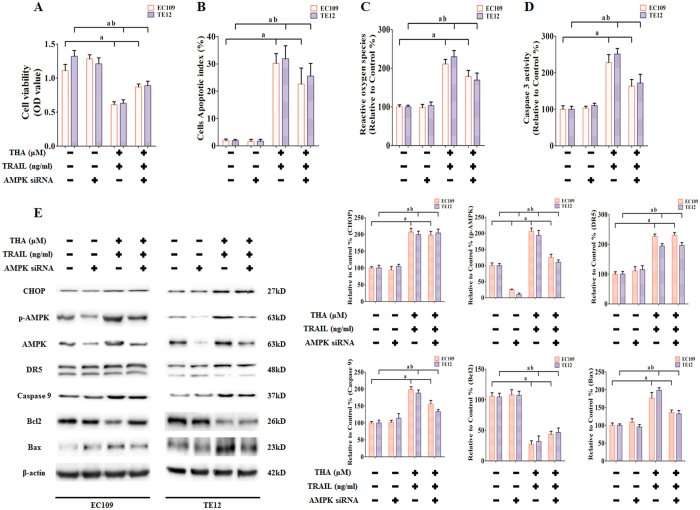
The role of AMPK production in thapsigargin-stimulated TRAIL-induced apoptosis in ESCC cells. After treated with AMPK siRNA for 48 h, the ESCC cells were administrated with thapsigargin (1 μM) and the TRAIL (100 ng/ml) for 24 h. **(A)** Viability is shown as OD values. **(B)** Apoptosis is shown as apoptotic index. **(C)** ROS concentrations are shown. **(D)** The intracellular Caspase 9 activity levels are shown. The two indexes in the control group were defined as 100%. **(E)** Representative Western blot results of the key proteins, including CHOP, p-AMPK, DR5, Caspase 9, Bcl2, and Bax are shown. Membranes were re-probed for β-actin expression to show that similar amounts of protein were loaded in each lane. The results are expressed as the mean ± SD; n = 6. ^**a**^P < 0.05 *vs.* the control siRNA group in EC109 cells, ^**ab**^P < 0.05 *vs.* the control siRNA group in TE12 cells.

Activation of AMPK signaling has been reported to be an important target in different diseases, including malignancy^18^. However, the role of AMPK phosphorylation in ESCC proliferation and survival is poorly understood. To investigate the activation of AMPK in thapsigargin-induced TRAIL sensitization, EC109 and TE12 cells were treated with Compound C (an AMPK-specific inhibitor) and AMPK siRNA to inhibit the AMPK phosphorylation and reduce AMPK production, respectively. Meanwhile the cells were co-treated with thapsigargin (1 μM) and the TRAIL (100 ng/ml) for 24 h. As shown in Figs 11E and 12E, Compound C and AMPK siRNA effectively decreased the phosphorylation and synthesis of AMPK. Moreover, Compound C and AMPK siRNA reversed thapsigargin-dependent sensitization to TRAIL in EC109 and TE12 cells, as evidenced by significantly increased cell viability (Figs 11A and 12A), inhibited apoptosis index (Figs 11B and 12B), reduced generation of ROS (Figs 11C and 12C), and decreased Caspase 3 activity (Figs 11D and 12D). However, Compound C and AMPK siRNA did not change the expression of CHOP and DR5, but did affect Caspase 9, Bcl2, and Bax expression, which indicated the relationship between these proteins. These findings confirmed that thapsigargin mediated sensitization to TRAIL in ESCC cell lines through the activation of AMPK from the aspects of protein function and existence.

### Combination of thapsigargin with hrTRAIL potentiates antiesophageal squamous cell carcinoma activity via AMPK phosphorylation *in vivo*

**Figure 13:**
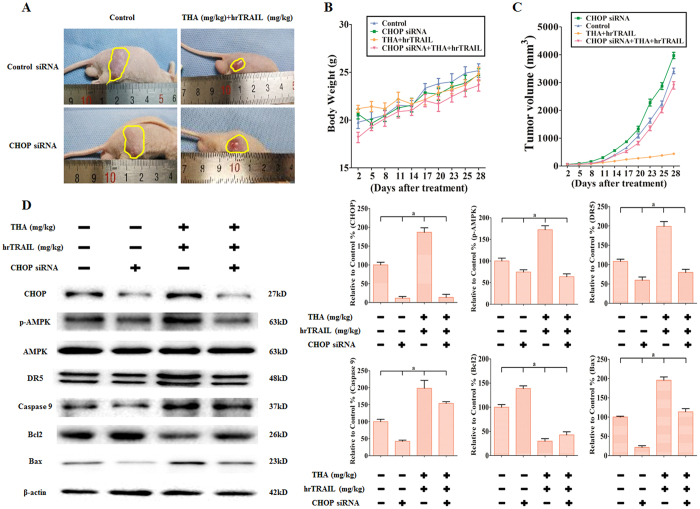
CHOP upregulation is critical for thapsigargin-stimulated TRAIL-induced apoptosis in nude mice carrying EC109 tumor xenografts. (**A**) Photographs showing tumor xenograft morphologies in each group. (**B**) Change in body weight of the mice. (**C**) A tumor growth curve was drawn from the tumor volumes and treatment duration. (**D**) Representative Western blot results of CHOP, p-AMPK, DR5, Caspase 9, Bcl2, and Bax are shown. Membranes were re-probed for β-actin expression to show that similar amounts of protein were loaded in each lane. The results are expressed as the mean ± SD; n = 6. ^a^P < 0.05 *vs.* the control group in EC109 cells.

EC109 cells were inoculated into the flanks of male nude mice. When tumors reached a mean group size of approximately 100 mm^3^, the mice were matched for tumor volumes and assigned to CHOP siRNA, thapsigargin and hrTRAIL co-treatment, or combination treatment with these three agents (Fig. 13A). Tumor volumes in the above groups were approximately 116.2%, 14.0%, and 84.7%, respectively, when compared with the control group (Fig. 13C). The combination of thapsigargin and the hrTRAIL significantly suppressed tumor growth not only when compared with the control group but also when compared with the group that received a combination of these three agents. Importantly, no substantial weight loss was observed in the mice in any treatment group during the period of therapy (Fig. 13B), indicating that thapsigargin, hrTRAIL, or CHOP siRNA were generally well tolerated *in vivo*. We next investigated the effect of the treatments on apoptosis *in vivo* by western blot analysis. As shown in Fig. 13D, thapsigargin and hrTRAIL co-treatment improved the expression of CHOP, increased AMPK phosphorylation, increased the expression of DR5, increased the expression of Bax, and activated Caspase 9, while suppressing Bcl2 expression. CHOP siRNA treatment not only inhibited the target protein but also antagonized the changes in the proteins mentioned above. Taken together, these data further suggest that the combination of thapsigargin with the hrTRAIL potentiated *in vivo* antitumor activity, which was in line with the *in vitro* results.

## Discussion

Esophageal cancer, an aggressive human malignancy, has steadily been increasing over the last 20 years worldwide in both males and females^25^. In the clinic, esophageal cancer is frequently diagnosed late, which results in poor outcomes. The potential curative therapeutic strategies including surgery, platinum-based chemotherapy, and radiotherapy are often ineffective and have high incidences of morbidity^8^. Moreover, systemic recurrence is common after curative-intent treatments^26^. Therefore, it is very important to explore the molecular basis of carcinogenesis, to elucidate the signal transduction pathways regulating cell growth and death and their roles in the process of malignant transformation, and to investigate novel molecularly targeted anticancer therapies.

The ER stress response pathway is a complex network that is responsible for maintaining ER homeostasis. It is initiated by 3 protein sensors, PERK, IRE1 and ATF6, each of which activates the ER stress response^27^. It has been implicated in cell physiological and pathological process, including carcinogenesis^28^. The PERK/ATF4/CHOP branch of the UPR plays an important role in cells undergoing ER stress^8^. Some studies have shown that the therapeutic induction of ERS-induced apoptosis might be beneficial for killing cancer cells^1^,^29^. TRAIL, as a member of the TNF family, can bind DR5/DR4 and induce apoptosis in many kinds of tumor cell lines *in vitro* and *in vivo*^10^,^30^. After binding its receptor, TRAIL activates caspase cascades^11^, and proapoptotic Bcl-2 family members, such as Bax and Bcl2, which accelerate intrinsic apoptosis^12^. CHOP is usually considered the main mediator of ER stress response-induced apoptosis and is a known transcription factor able to directly regulate DR5 expression^31^,^32^. Kim and colleagues showed that amiodarone could sensitize glioma cells to TRAIL-mediated apoptosis by CHOP-mediated DR5 upregulation^13^. Also, Chen *et al*. indicate that modulation of the unfolded protein response mediated by thapsigargin might be useful in sensitizing melanoma cells to TRAIL-induced apoptosis by up-regulation of TRAIL receptor^33^. Additionally, 20(S)-Ginsenoside Rg3 increased the protein level of DR5 and inhibited proliferation of human hepatocellular carcinoma cells, and this was regulated by CHOP^14^. Although the ER stress response has been previously implicated in the regulation of TRAILRs in many malignant diseases, the relationship of ER stress and TRAIL-mediated apoptosis, along with its downstream signaling molecules in human esophageal squamous cell carcinoma has not been critically elucidated. In our experiments, treatment with thapsigargin and the TRAIL resulted in dose-dependent induction of apoptosis and reduction in cell viability in EC109 and TE12 cells. Treatments also significantly decreased adhesion, migration, and invasion, all of which are major events related to tumor metastatic potential. Meanwhile, the anticancer effect of these two agents demonstrated synergistic action. Furthermore, thapsigargin effectively activated the ER stress response as evidenced by not only the upregulation of GRP78, CHOP, and ATF4 but also the increase of p-PERK and p-eIF2α levels in EC109 and TE12 cells. Simultaneously, thapsigargin could stimulate DR5 expression, which led to the sensitization of TRAIL treatment in ESCC cell lines. *In vitro* siRNA-mediated silencing of the ERS response protein CHOP, as well as DR5 siRNA, significantly reduced thapsigargin or TRAIL-induced cell death. Importantly, thapsigargin-induced activation of DR5 promoter activity was abrogated by mutation of the putative CHOP-binding site in the DR5 promoter. These data suggested that the anticancer activity of thapsigargin in human ESCC cell lines was mediated, at least in part, by the activation of ERS signaling and then upregulation of DR5 to sensitize the TRAIL treatment.

Caspase- and mitochondria-mediated apoptosis coordinately occur in response to a wide range of death stimuli^12^,^34^. Recently, activation of the Caspase 8 and Bcl-2 family pathway in ER-stress-induced or TRAIL-mediated apoptosis have been described^35^,^36^. For instance, cytotoxic chemotherapeutic drugs sensitize cultured cancer cells to TRAIL by alterations of the phenotypic expression of proapoptotic/antiapoptotic proteins that is paralleled with an overall increase of Caspase 8 activation and massive cell death^37^,^38^. The strong Caspase 8 activation may be secondary to activation by downstream caspases, such as Caspase 9, via Caspase 3^39^. Bax appears to play an important proapoptotic role, while Bcl2 is a critical inhibitory factor in this pathway of apoptosis^40^. In some situations, Bcl2 protects against apoptosis by reducing the release of Ca^2+^ from the ER and the translocation of the proapoptotic protein Bax to the mitochondria, thereby suppressing apoptosis^40^. Our results indicated that treatment with thapsigargin and the TRAIL dose-dependently downregulated Bcl2 expression, while upregulating Bax expression and Caspase 3 and Caspase 9 activity in EC109 and TE12 cells. In addition, our experiments confirmed that the inhibition of CHOP or DR5 with specific siRNAs reversed the effects of the above treatment on the expression of Bcl2 and Bax and on caspase activities. These results revealed that thapsigargin and the TRAIL synergistically suppressed the ESCC cells by the Caspase- and Bcl-2 family-mediated apoptosis pathway.

ROS play an important role in the determination of cancer cell fate, primarily in the mitochondria^41^. Recently, ROS have been identified as potential targets of novel anticancer drugs^42^. It has been shown that increased protein-folding load in the ER may result in the accumulation of ROS because increased protein synthesis would require increased disulphide bond formation in the ER, where electrons are shuttled through protein disulphide isomerase and endoplasmic reticulum oxidoreductin 1α (ERO1α) to O_2_ to generate H_2_O_2_^43^. Nicotinamide adenine dinucleotide phosphate (NADPH), a vital multi-enzyme complex in redox signal transduction for cell growth and apoptosis, is responsible for ROS generation^44^. In our study, thapsigargin activated the ER stress associated with a rapid increase in intracellular ROS levels and NADPH oxidase activity in EC109 and TE12 cells. Furthermore, inhibition of ERS with CHOP siRNA or desensitization of TRAIL with DR5 siRNA attenuated thapsigargin- and TRAIL-induced ROS production and NADPH oxidase activity. GSH is a major antioxidant of intracellular ROS that affects cell activity more sensitively in nonmalignant cells than in malignant cells^45^. The extent of exposure to ROS and of perturbation of the GSH redox balance play critical roles in determining whether cells undergo pro-survival or pro-death processes. In the present study, thapsigargin and TRAIL-induced treatments synergistically decreased the consumption of intracellular GSH, and this was further accompanied by increased ROS. These effects were reversed by specific siRNA intervention. Based on the above findings, we suggest that thapsigargin sensitizes human esophageal cancer to TRAIL-induced apoptosis via exhausting the total cellular antioxidant capacity and increasing the ROS levels in human ESCC cells.

AMPK acts as an important sensor of intracellular energy levels and is activated in response to metabolic stress to mediate energy balance, because it monitors the AMP/ATP ratio to regulate cellular metabolism by restoring ATP levels^16^,^46^,^47^,^48^,^49^. Some reports indicate the ability of AMPK to induce apoptosis of neuroblastoma cells^50^, pancreatic cells^51^, and gastric cancer cells^52^. These studies suggest that AMPK signaling might facilitate growth inhibition and cell killing, serving as a new therapeutic target in cancer diseases^18^. Importantly, Li and colleagues found that the ratio of phosphorylated AMPK to total AMPK was much lower in ESCC compared with adjacent normal tissue, which indicated that AMPK activators may be potentially useful in cancer therapy^53^. Furthermore, many studies demonstrated that both ER stress and the TRAIL could play efficient roles against cancer via the AMPK pathway. ER is the main storage site of Ca^2+^, and any disruption in its accumulation can also promote ER stress^43^. Thapsigargin causes ER calcium depletion through irreversible inhibition of the ER Ca^2+^-ATPase pump which was responsible for the influx of Ca^2+^ from cytosol^54^. The increased cytoplasmic calcium activates a calcium signaling pathway which further phosphorylated AMPK to trigger apoptosis and autophagy^55^. For instance, Yoo and colleagues reported that melatonin regulates insulin synthesis through the endoplasmic reticulum (ER) via human antigen D (HuD) expression in rat insulinoma cells, and this was mediated by the activation of AMPK^56^. As mentioned above, TRAIL, a member of the TNF family, is considered a promising anticancer agent due to its ability to induce apoptosis in a variety of tumor cell types while having only negligible effects on normal cells^57^,^58^. TNFα, the best characterized necrosis-inducing ligand, has a direct involvement on mitochondrial ATP production^59^. According to Warburg’s work that cancer cells mainly use glycolysis for energy metabolism in either the presence or absence of oxygen^60^. Thus, it is reasonable to believe that glucose metabolism or ATP production could be the key role in TRAIL-induced apoptosis^61^. Because, once activated by energy stress, phosphorylation of AMPK acts to restore energy homeostasis by promoting catabolic pathways generating ATP^62^,^63^. In other word, TRAIL-induced apoptosis consumes large amounts of ATP in cell, which actives the AMPK pathway. For example, Su *et al*. discovered that the potentiation of TRAIL- and TNFα-induced tumor cell apoptosis by AICAR (an AMPK-specific agonist) is dependent on AMPK and p53 and involves various aspects of apoptotic pathways^12^. However, the role of AMPK in the anti-ESCC activity of ER stress-induced sensitization of TRAIL has not been elucidated thoroughly. In this study, we concomitantly used thapsigargin and TRAIL as cytotoxic inducers to elucidate their combinatorial effects under the condition of inhibition of AMPK with Compound C and AMPK siRNA. The results showed that the synergetic cell cytotoxicity induced by thapsigargin and TRAIL in EC109 and TE12 cells was abrogated by Compound C and AMPK siRNA, as reflected by promoting cell survival, suppressing cell apoptosis, reducing ROS production, and decreasing Caspase 3 activity. Meanwhile, Compound C and AMPK siRNA also inhibited the activation of Bcl-2 family proteins (Bcl2 and Bax) and the expression of Caspase 9, both of which were stimulated by thapsigargin and TRAIL co-treatment, but did not affect CHOP and DR5. These results confirm the existence of thapsigargin-induced and TRAIL-induced regulatory pathways that promote apoptosis via AMPK activation from the aspects of protein function and existence.

RNAi-based approaches have been used as therapeutic methods for the treatment of a variety of tumor types^64^,^65^. In the present study, we injected CHOP-siRNA directly into the abdominal cavities of nude mice burdened by ESCC cell xenografts and found that, at day 20 after the first injection, the mean tumor volume of the tested group was increased by 38.63% compared with the control. Considering that CHOP correlated with patient survival and remained an independent prognostic variable in some cancers, it may be useful in patient stratification and the development of anticancer strategies aimed at modulating ER stress^66^,^67^. Based on our study, in response to the combined intraperitoneal injection of CHOP-siRNA along with administration of thapsigargin and the TRAIL, the inhibition of tumor growth was inferior to that of thapsigargin and TRAIL co-treatment. Moreover, western blot analysis showed that the upregulation of DR5 and the activation of p-AMPK and Caspase 9 induced by thapsigargin and TRAIL co-treatment were weakened by CHOP siRNA, which was in agreement with the *in vitro* results.

**Figure 14:**
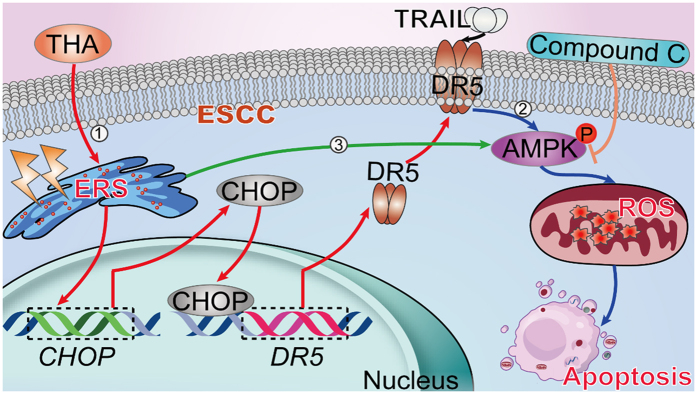
Schematic diagram of thapsigargin-stimulated TRAIL-induced apoptosis in human ESCC. ER stress response could effectively sensitize human esophageal cancer to TRAIL-mediated apoptosis via TRAILRs and AMPK pathway. Details are as follows: ① Induction of ER stress with thapsigargin increases the CHOP expression, and then CHOP upregulates DR5 by combining to CHOP-binding site. Upregulation of DR5 improves the receptor number which could bond more TRAIL in ESCC cells. ② TRAIL/DR5 binding induced ESCC cells apoptosis mediated by ROS via AMPK phosphorylation. ③ Induction of ER stress can directly activate AMPK phosphorylation, which play an efficient role against ESCC cells. ESCC, esophageal squamous cell carcinoma; THA, thapsigargin; ERS, endoplasmic reticulum stress; CHOP, C/EBP-homologous protein; DR5 (i.e., Apo2), death receptor 5; TRAIL, tumor necrosis factor (TNF)-related apoptosis-inducing ligand; AMPK, adenosine monophosphate activated protein kinase; ROS, reactive oxygen species.

In conclusion, our results suggest a direct role of the ER stress response in effectively sensitizing human esophageal cancer to TRAIL-mediated apoptosis, meanwhile implicating TRAILRs and the AMPK pathway in this process (Fig. 14). The following is a detailed explanation: ① Induction of ER stress with thapsigargin increases CHOP expression, thus enhancing DR5 upregulation. ② TRAIL/DR5 activation induces ESCC cells apoptosis via AMPK phosphorylation triggered oxidative stress and cell apoptosis. ③ Induction of ER stress can also directly activate AMPK phosphorylation, which promotes ESCC cells apoptosis. Therefore, our results suggest that the activation of ER stress may be beneficial for improving the efficacy of TRAIL-based cancer therapy.

## Materials and Methods

### Cell culture

EC109 and TE12 cells (two human ESCC cells lines) were provided by the cell bank of type culture collection of Chinese academy of sciences (Shanghai Institute of Cell Biology, Chinese Academy of Sciences, Shanghai, China). HET-1A cell (immortalized human primary esophageal epithelial cell) was purchased from American Type Culture Collection (Manassas, Virginia, USA). The ESCC cells and HET-1A cell were routinely cultured in RPMI-1640 medium (BioWhittaker, Walkersville, MD, USA) at 37 °C in a humidified atmosphere containing 5% CO_2_. The media were supplemented with 10% fetal bovine serum, penicillin (100 units/ml), and streptomycin (100 units/ml) (Invitrogen, CA, USA) according to the provider’s instructions^8^.

### Reagents

Thapsigargin (an ERS inducer), 3-(4,5-dimethylthiazol-2-yl)-2,5-diphenyltetrazolium bromide (MTT), dimethyl sulfoxide (DMSO), 2′,7′-dichlorofluorescein diacetate (DCFH-DA), 4′,6-diamidino-2-phenylindole (DAPI), trypsin, and protease inhibitor cocktail were purchased from Sigma Chemical Corporation (St. Louis, MO, USA). Commercial TRAIL (19.6 kDa protein, amino acid 168) was from KOMA Biotech (Yeongdeungpo-gu, Seoul, Korea) for cell experiment. Human recombination TRAIL (hrTRAIL) was designed according to previous study and synthesized by Hanbio Biotechnology Co., Ltd (Shanghai, China) for animal experiment. Compound C (6-[4-(2-piperidin-1-yl-ethoxy)-phenyl]-3-pyridin-4-ylpyrrazolo[1,5-a]-pyrimidine), an AMPK inhibitor, was purchased from Millipore (Merck, Billerica, MA, USA). The following antibodies were used: anti-caspase 3, anti-caspase 9, and anti-Bcl2; anti-Bax, anti-AMPK, anti-phospho-eIF2a (p-eIF2α), anti-eIF2α, anti-CHOP, anti-ATF4, and anti-β-actin (Cell Signaling Technology, Beverly, MA, USA); anti-DR5 (KOMA Biotech, Yeongdeungpo-gu, Seoul, Korea); anti-GRP78, anti-phospho-PERK (p-PERK), and anti-PERK (Santa Cruz Biotechnology, Santa Cruz, CA, USA); horseradish peroxidase-conjugated anti-rabbit immunoglobulin G (IgG) and horseradish peroxidase-conjugated anti-mouse IgG (Invitrogen, Carlsbad, CA, USA). Terminal deoxynucleotidyl transferase dUTP nick-end labeling (TUNEL) kits were purchased from Roche Diagnostics (Mannheim, Germany). Caspase 3 and Caspase 9 colorimetric assay kit were purchased from R&D Systems (Minneapolis, MN, USA). A glutathione (GSH) assay kit was obtained from Shanghai Enzyme-linked Biotechnology Co., Ltd. (Shanghai, China).

### Small Interfering RNAs

The siRNA sequences against human CHOP, DR5, and nonsilencing were chemically synthesized by Genepharma (Shanghai, China). siRNA sequences were as follows: CHOP siRNA: UUCAUCUGAAGACAGGACCUCUUGC, DR5 siRNA: AUCAGCAUCGUGUACAAGGUGUCCC, Nonsilencing siRNA: TTCTCCGAACGTGTCACGT. The commercial AMPK and control siRNA were purchased from Santa Cruz Biotechnology (Santa Cruz, CA, USA). Concretely, EC109 and TE12 cells were plated on 6-well plates at 3 × 10^5^ cells per well and transfected with 100 pmol of siRNA duplex per well using Lipofectamine 2000 (Invitrogen) as the manufacturer’s recommendations. Cells were harvested 48 h after transfection.

### Cells treatments

Thapsigargin was dissolved in DMSO, which was diluted in culture media immediately prior to an experiment. The concentration of DMSO was less than 0.1%. FBS-free RPMI-1640 containing same volume of DMSO as above was used as the control. The ESCC cells were treated with thapsigargin (1 and 2 μM) and TRAIL (100 and 200 ng/ml) for 24 h in the first part of our study; and then in the absence or presence of CHOP siRNA, DR5 siRNA, or AMPK siRNA (pretreated for 24 h) cells were treated with thapsigargin (1 μM) and TRAIL (100 ng/ml) for further 24 h in the second part of our study; at last, cells were simultaneously treated with thapsigargin (1 μM), TRAIL (100 ng/ml), and Compound C (1 μM) for 24 h. After the different treatments, the cells were harvested for further analysis. The HET-1A cells was administrated with thapsigargin (1 and 2 μM) and TRAIL (100 and 200 ng/ml), similarly.

### Terminal deoxynucleotidyl transferase-mediated dUTP nick end labeling

EC109 and TE12 cell apoptosis was analyzed using an *In Situ* Cell Death Detection Kit according to the manufacturer’s instructions, and the results were obtained using a fluorescence microscope. Briefly, the ESCC cell lines grown on cover slips were washed twice with PBS and fixed in 4% paraformaldehyde for 30 min; subsequently, endogenous peroxidase activity was inhibited by incubation in 3% hydrogen peroxide diluted in methanol for 10 min at room temperature. After permeabilized using 0.1% Triton X-100 for 5 min on ice, the cells were incubated with 50 μL of the TUNEL reaction mixture. Two negative controls were generated using 50 μL of Label Solution. Finally, the nuclei were stained with DAPI-containing mounting medium prior to visualization using a BX51 light microscope (Olympus, Japan). The TUNEL-positive cells were counted in 5 randomly selected fields under high-power magnification. The apoptotic index was expressed as the ratio between the number of TUNEL-positive cells and the total number of DAPI-positive cells.

### Cytotoxicity assay

Cell viability was determined using tetrazolium colorimetric tests (MTT test). Optical density (OD) values were obtained at 570 nm using a microplate reader (SpectraMax 190, Molecular Device, USA), and cell viability was expressed as the OD value. Cell morphological images were taken by phase contrast microscopy with a 600D camera (Canon Company, Japan). Presented data were from representative experiments of at least 3 independent assays.

### Cell matrigel invasion

Cells were seeded on a fibronectin-coated polycarbonate membrane insert in a Transwell apparatus (Costar, Flintshire, UK). The Transwell membrane was coated with 300 ng/ml Matrigel (BD Biosciences, San Jose, CA, USA)RPMI 1640 containing 20% fetal bovine serum (Invitrogen) was added to the lower chamber. After incubation for different treatment for the indicated time, the insert was washed with phosphate-buffered saline, and cells on the top surface of the insert were removed by wiping with a cotton swab. The cells on the lower surface were stained with 0.4% crystal and counted in three random fields at x200 magnification.

### Adhesion analysis and wound-healing assay

We performed adhesion and migration analysis with lower concentrations of agents which do not affect cell activity. In our preliminary experiments, we found that Thapsigargin (lower than 0.6 μM) and TRAIL (lower than 70 ng/ml) for 24 h did not affect the proliferation of the two ESCC cells lines (Data were not shown).

After treatment, 100 μL of medium containing 1 × 10^4^ cells was incubated to for 0.5 h in a 96-well plate. The medium in each well was then discarded for adhesion analysis. The adherent cells were stained with MTT. The stained cells were observed using an inverted phase-contrast microscope. Images were acquired using a 600D camera (Canon, Japan), and five fields were randomly selected for quantification. Finally, 100 μL of DMSO was added to each well, and the plates were incubated for 30 min at 37 °C with shaking. The OD value of each well at 570 nm was measured using a SpectraMax 190 spectrophotometer (Molecular Devices, Sunnyvale, CA, USA), and the OD value of the control group was normalized to 100%.

For wound-healing assay, cells were seeded into 60-mm dishes. When the cells were grown to confluence, three scrape wounds were made for each sample with a P200 pipette tip. The wound edge movement was then photographed under a microscope, and the results were expressed as the distance between the sides of the scratch. The distance in the control group was set to 100%.

### Measurement of reactive oxygen species (ROS), NADPH oxidase activity, and GSH level

After treatment, samples were trypsinized and subsequently incubated in DCFH-DA (5 μM) in PBS for 2 h at 37 °C. The DCFH fluorescence of samples were measured using an FLX 800 fluorescence microplate reader at an excitation of 488 nm and an emission of 522 nm (Biotech Instruments, Inc., USA). A cell-free measurement was used as the background, and the fluorescence intensity in the control group was defined as 100%.

The NADPH oxidase activity assay was performed by modifying the method described by previous study^68^. Briefly, the specimens were harvested in Krebs-HEPES buffer (pH 7.4) containing protease and phosphatase inhibitor cocktail, homogenized, and centrifuged at 10,000 × g for 15 min. Equal amount of proteins was added with 10 μM lucigenin prepared in the same buffer and incubated at 37 °C for 10 min. Over the subsequent 1 min, the chemiluminescence was measured in response to the addition of 100 μM NADPH using the luminescence channel of a Fluostar Optima microplate reader (BMG Labtech, Offenburg, Germany). Data are expressed as relative levels in the control group and the NADPH oxidase activity in the control group was normalized to 100%.

The generation of intracellular reduced GSH was simultaneously measured using commercial kits according to the provided instructions. Briefly, mitochondria were lysed in the presence of iodoacetic acid (10 mM) and were derivatized with fluorescent dansyl chloride. The derivatized samples were separated and analyzed via hydrophilic interaction liquid chromatography using a Restek Ultra Amino 3-μm 100 × 3.2 mm HPLC column at a flow rate of 0.6 mL/min. Buffer A (80% methanol/20% water) was mixed in a linear gradient of up to 30% of buffer B [acetate-buffered (pH 4.6) methanol solution] to elute GSH using a Varian ProStar 230 HPLC system. Fluorescent products were measured using an FLX 800 fluorescence microplate reader at an excitation of 335 nm and an emission of 541 nm. The GSH level in the control group was normalized to 100%.

### Analysis of Caspase 3 and Caspase 9 activities

Caspase 3 and Caspase 9 activities were measured using a colorimetric assay kit according to the manufacturer’s instructions. The cell and tissue lysates (20 μl) were added to a buffer containing a p-nitroaniline (pNA)-conjugated substrate for Caspase 3 (Ac-DEVD-pNA) and Caspase 9 (LEHD-pNA) to yield a 100-μl reaction volume. The reactions were performed at 37 °C for 2 h. The released pNA concentrations were calculated based on the absorbance values at 405 nm and the calibration curve of the defined pNA solutions. The Caspase 3 and Caspase 9 activities in the control group were set as 100%.

### Luciferase assay

The pDR5-WT [containing the DR5 promoter sequence (−605/+3)] and pDR5-mCHOP (containing a point mutation in the CHOP binding site to DR5/−605) were transfected into ESCC cells. After 24 hours, transfected cells were treated with or without thapsigargin (1 μM) plus the TRAIL (100 ng/ml) for additional 24 hours, and cell lysates were analyzed for luciferase activity following the manufacturer’s protocol (Promega, Madison, WI, USA).

### Western blot analysis

Western blot analysis was performed utilizing procedures described previously^69^. Briefly, equal amounts of total cells and tissues extracts were fractionated by SDS-PAGE, transferred to PVDF membranes, and subjected to western blot analysis with various antibodies. The fluorescent signal was detected using a BioRad imaging system (BioRad, Hercules, CA, USA), and the signal was quantified using Image Lab Software (BioRad, Hercules, CA, USA).

### Xenograft assays in nude mice

Male athymic nude mice were purchased from the Laboratory Animal Centre of the Fourth Military Medical University. The mice were fed and maintained under specific pathogen-free conditions in facilities approved by the American Association for Accreditation of Laboratory Animal Care and in accordance with the current regulations and standards of the United States Department of Agriculture and the United States Department of Health and Human Services. All the operation procedures in terms of cell lines and animals were carried out in accordance with the Institutional Animal Care and Use Committee guidelines and were approved by the Institutional Animal Ethics Committee of the Fourth Military Medical University (Permit No. 16001, 16002). Nude mice were s.c. injected with 7 × 10^6^ EC109 cells into both flanks of each animal, respectively. The body weight and tumor size of each mouse were measured every 3 days. On day 28, the tumors were excised from euthanized mice for additional analysis. The diameter of the tumors was measured using a vernier caliper. The estimated tumor volume was calculated using the formula: volume = 0.5 × length × width^2^.

### Anticancer activity in a xenograft model

The invivofectamine-Control-siRNA complex and invivofectamine-CHOP-siRNA complex were prepared according to the manufacturer’s instructions (Life Technology, Carlsbad, CA, USA). The stock solution, 250 μl, containing siRNA 3 mg/ml was mixed with isovolumetric complex buffer. Invivofectamine reagent (500 μl) was added to the mixture, and the mixture was incubated at 50 °C for 0.5 h. The mixture was then added to the Float-A-Lyzer dialysis device (Thermo Scientific, Waltham, MA, USA) and incubated for 2 h at room temperature in 1 L of phosphate-buffered saline^70^.

After the tumor volume was approximately 100 mm^3^, the mice were randomly divided into 4 different treatment groups treated with the following different therapies: (1) 0.9% normal saline (NS); (2) NS + CHOP siRNA (100 μl); (3) thapsigargin (1 mg/kg) + hrTRAIL (60 mg/kg); (4) thapsigargin (1 mg/kg) + hrTRAIL (60 mg/kg) + CHOP siRNA (100 μl). All of the agents were administered intraperitoneally, five times per week^30^,^71^.

### Statistical analyses

All of the data are presented as the means ± standard deviation (m ± SD). When comparing multiple groups, one-way ANOVA followed by Bonferroni post hoc test was performed. A P value of less than 0.05 was considered to be significant.

## Additional Information

**How to cite this article**: Ma, Z. *et al*. Thapsigargin sensitizes human esophageal cancer to TRAIL-induced apoptosis via AMPK activation. *Sci. Rep.* **6**, 35196; doi: 10.1038/srep35196 (2016).

## Supplementary Material

Supplementary Information
