# Supplementary material for: Thapsigargin sensitizes human esophageal cancer to TRAIL-induced apoptosis via AMPK activation
Source: Sci Rep. 2016 Oct 12;6:35196. doi: 10.1038/srep35196 (PMC5059685; doi:10.1038/srep35196)

Supplementary Information

Title of manuscript:

Thapsigargin sensitizes human esophageal cancer to TRAIL-induced apoptosis via AMPK activation

Authors:

Zhiqiang Ma, Chongxi Fan, Yang Yang, Shouyin Di, Wei Hu, Tian Li, Yifang Zhu, Jing Han, Zhenlong Xin, Guiling Wu, Jing Zhao, Xiaofei Li, and Xiaolong Yan

Supplementary information includes:

Supplementary Figs. S1-S8

Supplementary Fig. 1: The effects of Low concentrations of Thapsigargin and TRAIL on the viability and phosphorylation of AMPK in human ESCC cells. Human ESCC cells (EC109 and TE12) were treated with relatively low concentrations of thapsigargin (0.3 and 0.6 μM) and TRAIL (35 and 70 ng/ml) for 24 h. The cell viability **(A)** and phosphorylation of AMPK **(B)** were analyzed by MTT and western blot, respectively. The cell viability is expressed as OD values. All of the results are expressed as the mean ± SD.

Supplementary Figure 1

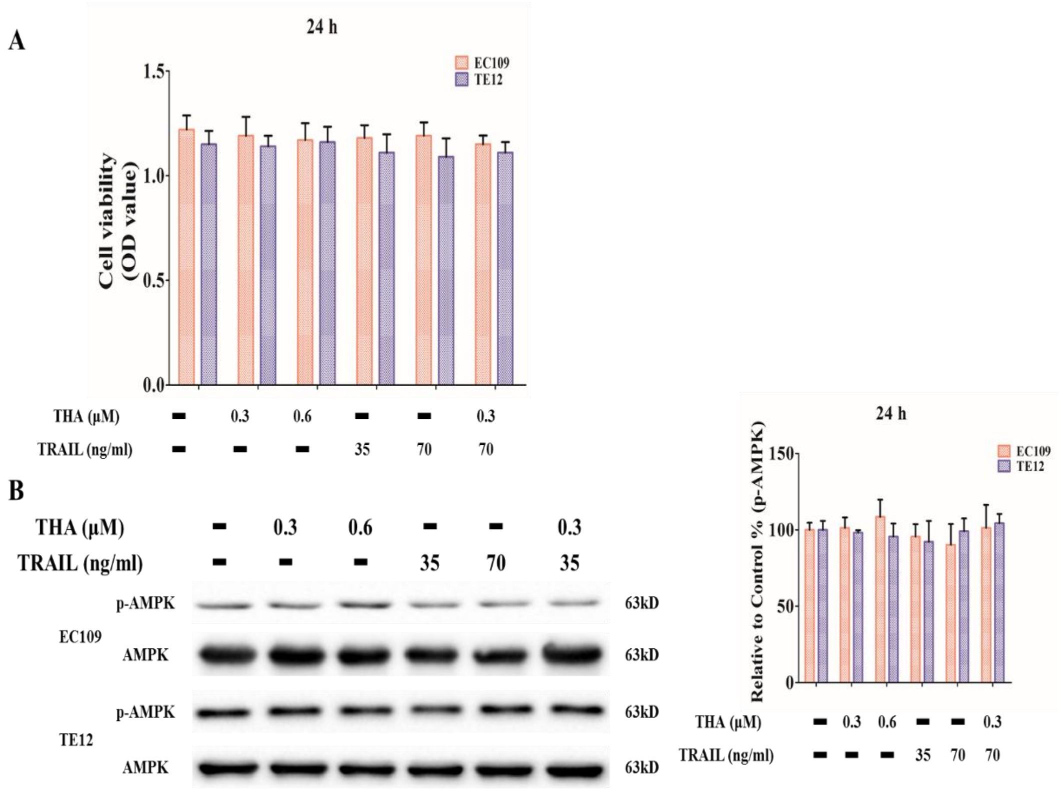

Supplementary Fig. 2: The full length blots in Fig. 7

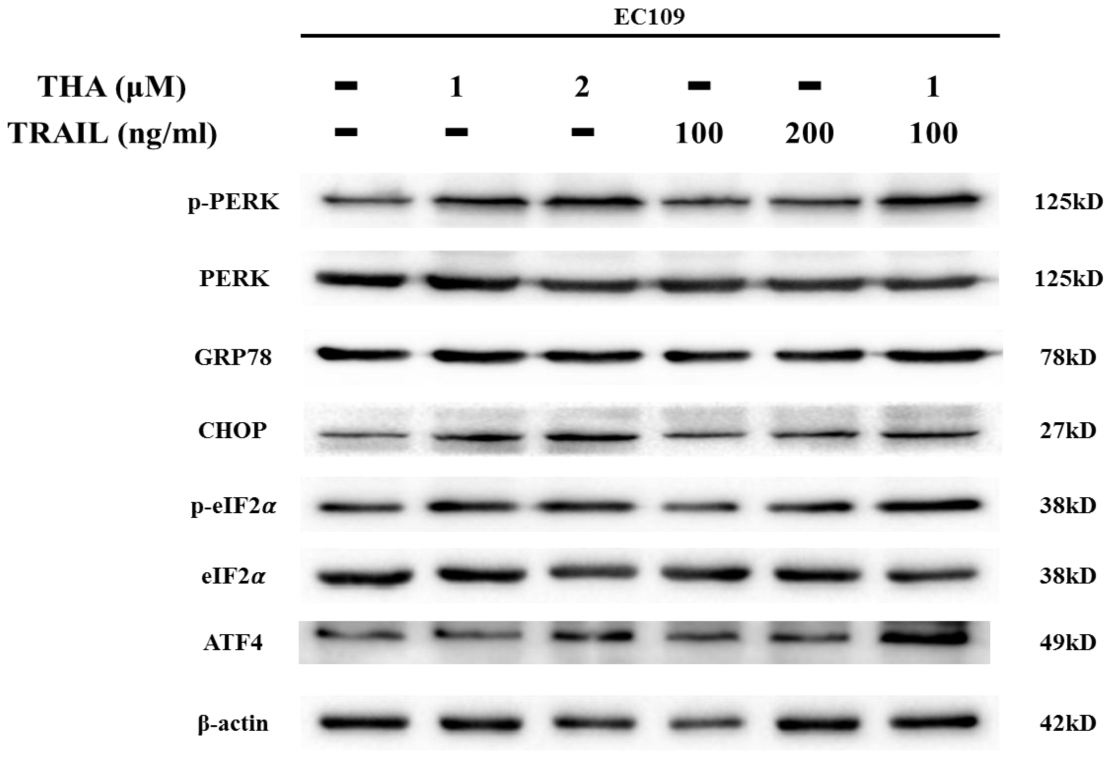

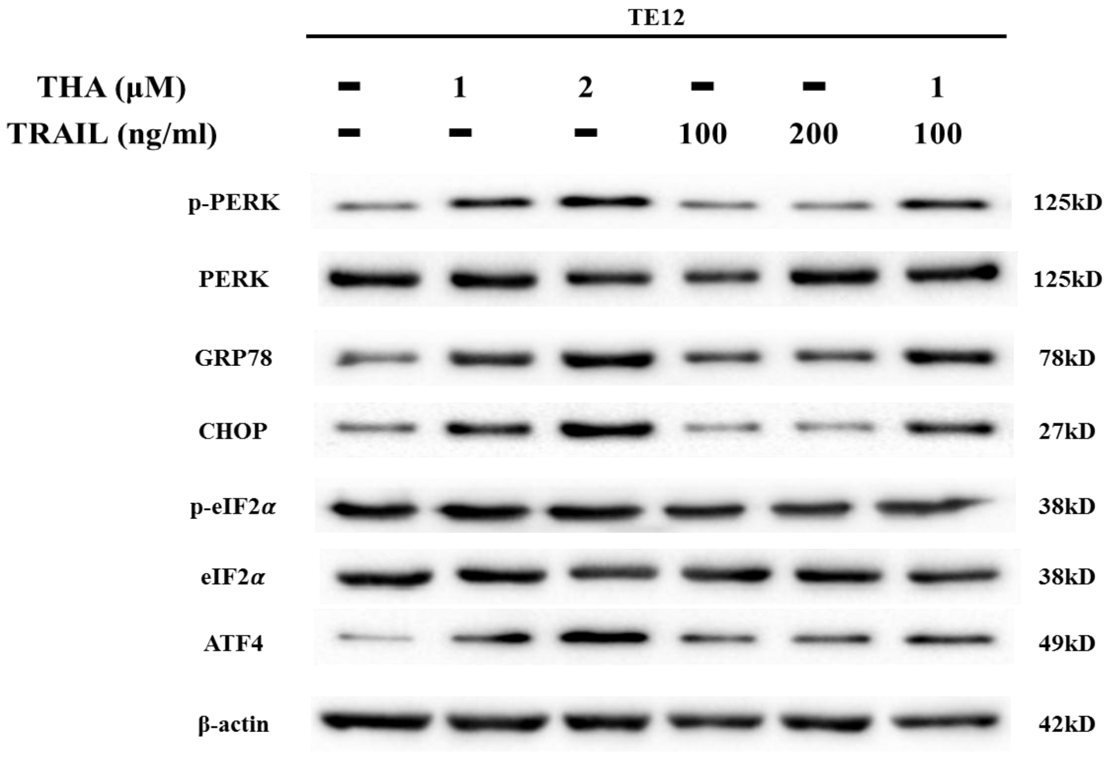

Supplementary Fig. 3: The full length blots in Fig. 8

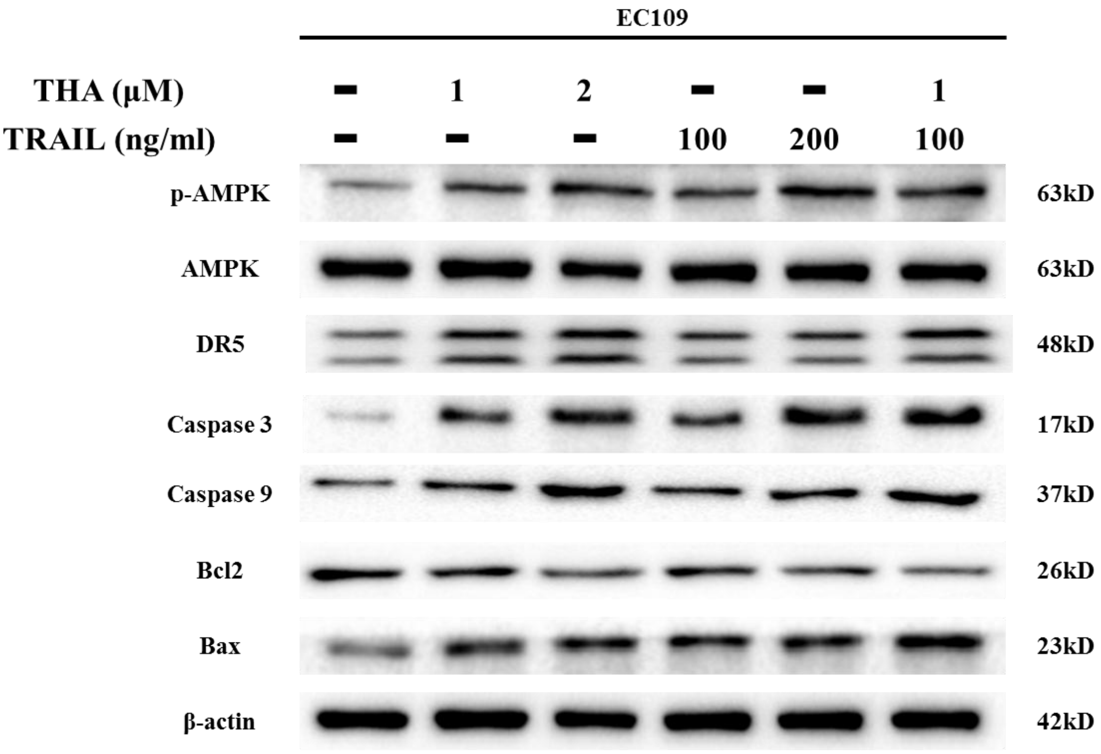

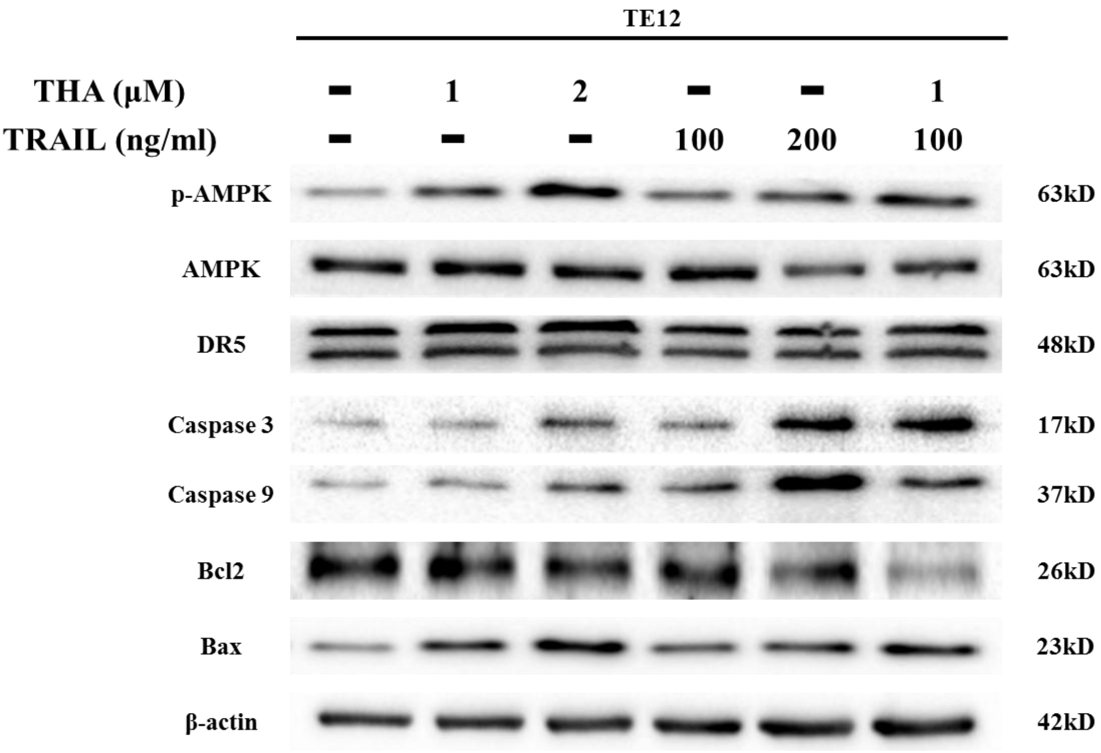

Supplementary Fig. 4: The full length blots in Fig. 9E

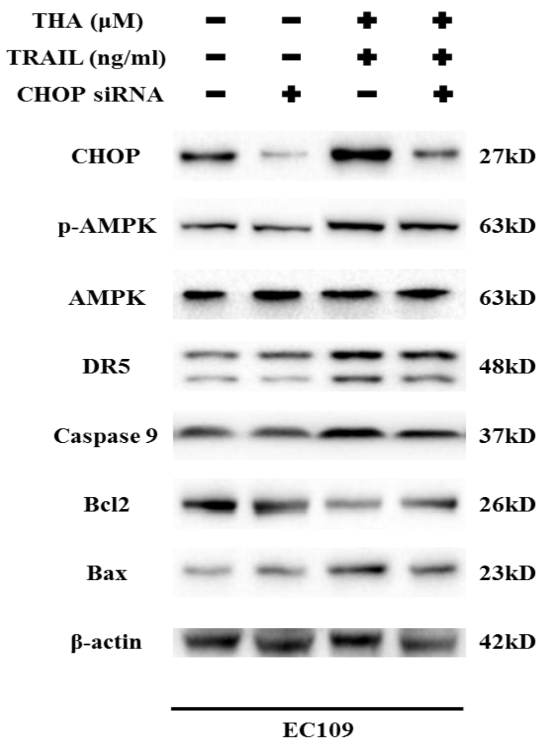

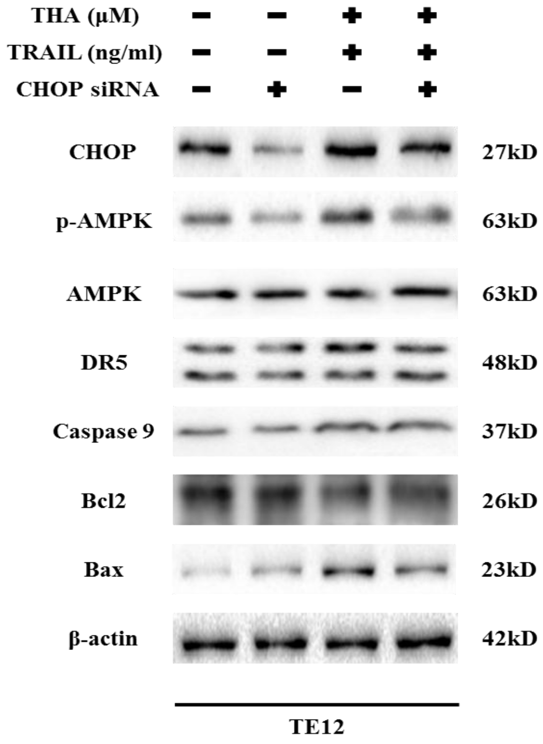

Supplementary Fig. 5 The full length blots in Fig. 10E

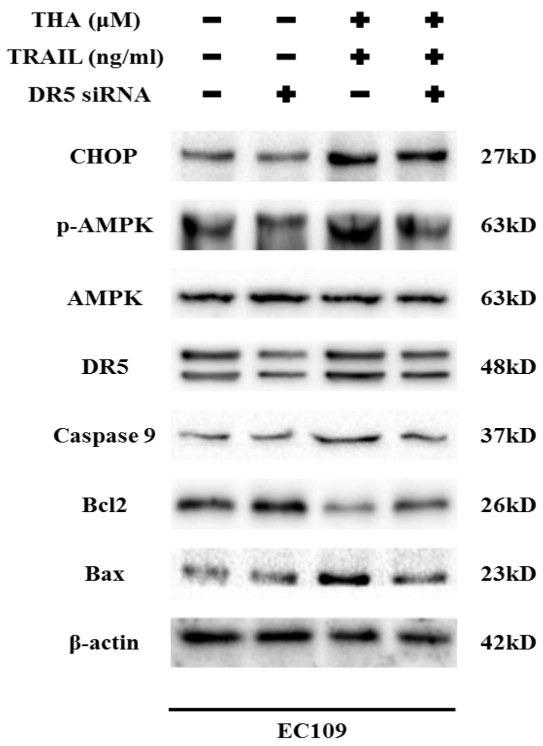

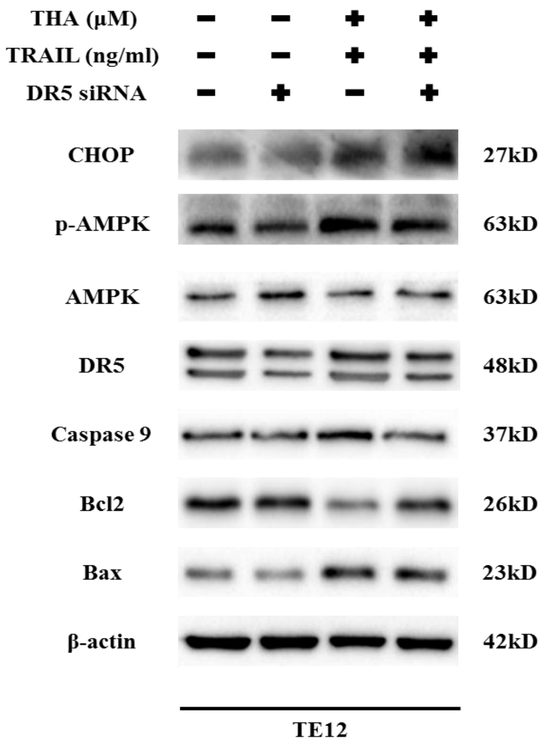

Supplementary Fig. 6 The full length blots in Fig. 11E

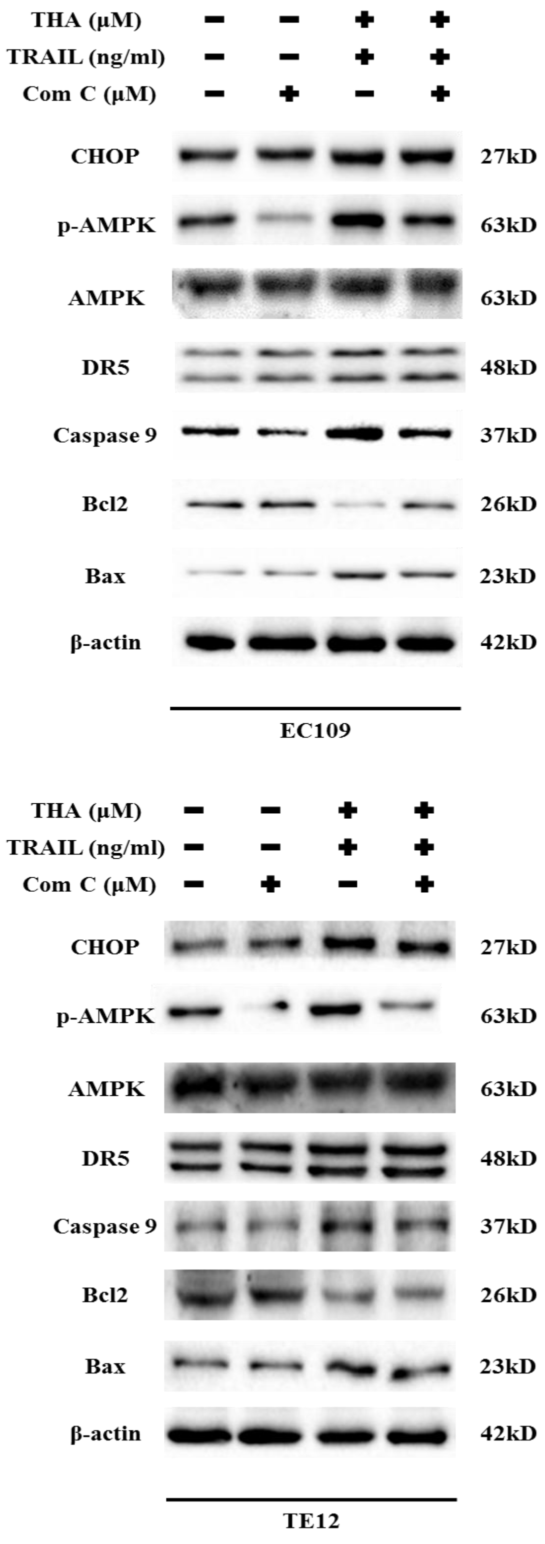

Supplementary Fig. 7 The full length blots in Fig. 12E

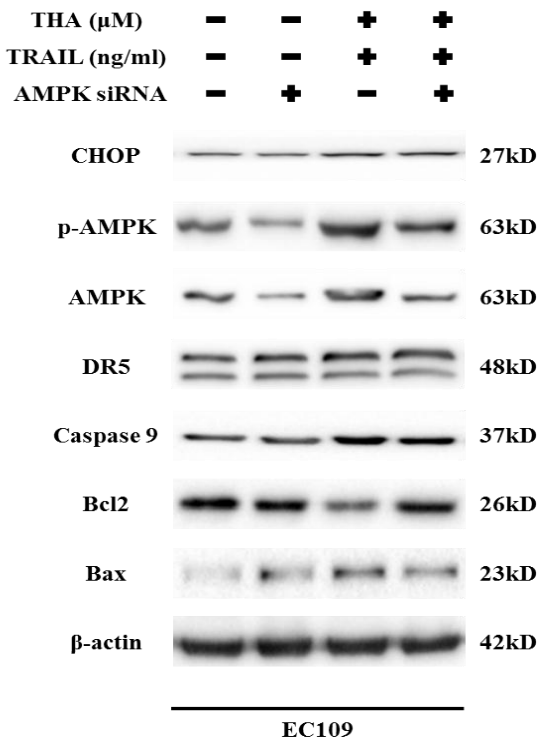

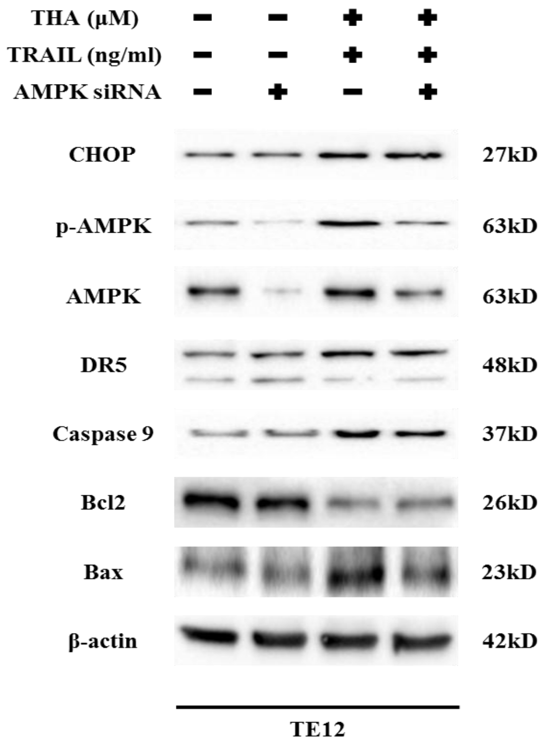

Supplementary Fig. 8 The full length blots in Fig. 13D

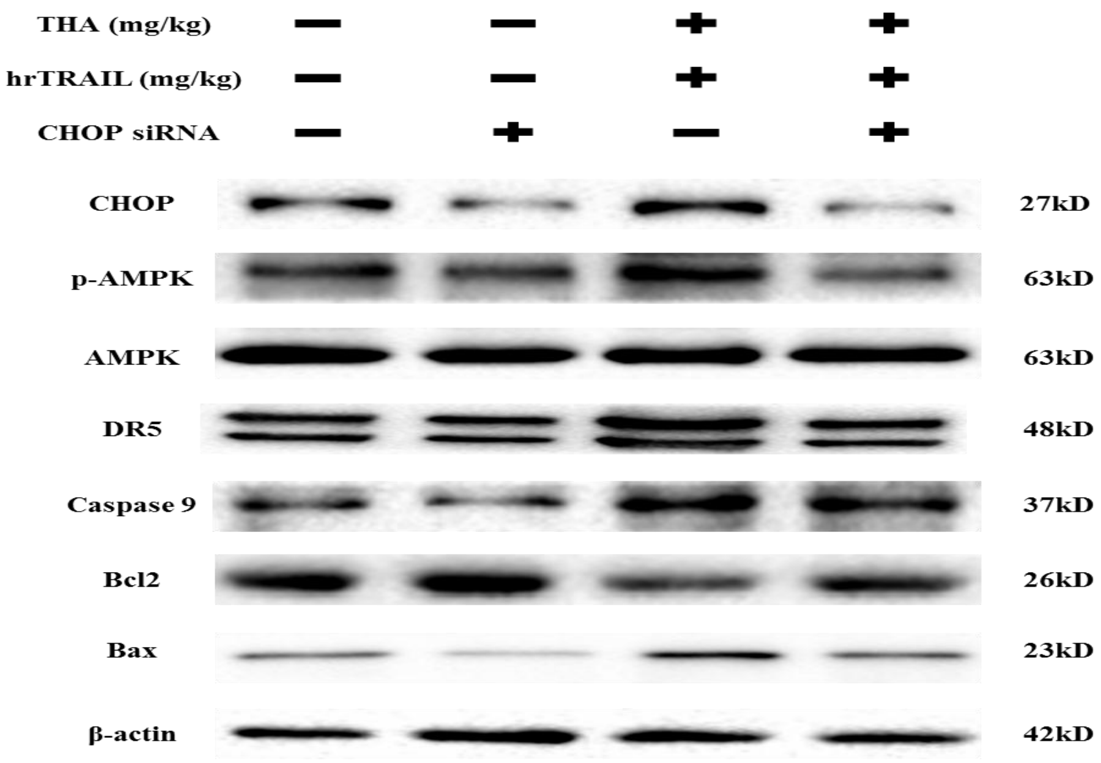

Supplement: Supplementary Information [file srep35196-s1.pdf]
